# Endocan Knockdown Down-Regulates the Expression of Angiogenesis-Associated Genes in Il-1ß Activated Chondrocytes

**DOI:** 10.3390/biom13050851

**Published:** 2023-05-18

**Authors:** Michele Scuruchi, Federica Aliquò, Angela Avenoso, Giuseppe Mandraffino, Giovanna Vermiglio, Aurelio Minuti, Salvatore Campo, Giuseppe Maurizio Campo, Angela D’Ascola

**Affiliations:** 1 Department of Clinical and Experimental Medicine, University of Messina, 98122 Messina, Italy; 2 Department of Biomedical and Dental Sciences and Morphofunctional Images, University of Messina, 98122 Messina, Italy

**Keywords:** endocan, proteoglycans, chondrocytes, inflammation, angiogenic factors

## Abstract

Endocan is a small soluble proteoglycan (PG) known to be involved in inflammation and angiogenesis. Increased endocan expression was found in the synovia of arthritic patients and chondrocytes stimulated with IL-1ß. Considering these findings, we aimed to investigate the effects of endocan knockdown on the modulation of pro-angiogenic molecules expression in a model of IL-1ß-induced inflammation in human articular chondrocytes. Endocan, VEGF-A, MMP-9, MMP-13, and VEGFR-2 expression was measured in both normal and endocan knockdown chondrocytes stimulated with IL-1ß. VEGFR-2 and NF-kB activation were also measured. Results have shown that endocan, VEGF-A, VEGFR-2, MMP-9, and MMP-13 were significantly up-regulated during IL-1ß-induced inflammation; interestingly, the expression of such pro-angiogenic molecules and NF-kB activation were significantly reduced by endocan knockdown. These data support the hypothesis that endocan released by activated chondrocytes may be involved in the mechanisms that stimulate cell migration and invasion, as well as angiogenesis, in the pannus of arthritic joints.

## 1. Introduction

Angiogenesis is a physiological process consisting of the growth of new blood vessels from the existing ones [1]. Physiological angiogenesis plays a key role in several physiological processes, such as growth and development, tissue regeneration, and repair; on the contrary, imbalanced angiogenesis is found in several pathological conditions including cancer, where it promotes tumor progression and metastasis, and arthritis, where it is accompanied by catabolic events that lead to cartilage joint destruction [2,3].

As known, articular cartilage is a highly specialized connective tissue composed of a complex network of extracellular matrix (ECM) molecules and chondrocytes. Unlike other tissues, adult cartilage, in physiological conditions, is devoid of vascular structures, and the expression of pro-angiogenic factors is down-regulated in chondrocytes [2,3,4]. 

On the contrary, during degenerative joint diseases, like osteoarthritis (OA) and rheumatoid arthritis (RA), hypertrophic chondrocytes promote angiogenesis through the expression of factors controlling endothelial cell migration, invasion, and adhesion, thus contributing to disease progression [5]. In this setting, evidence has shown that VEGF, the most potent pro-angiogenic factor, is up-regulated and associated with catabolic processes in both synovial cells and chondrocytes [6,7,8,9,10,11]. Considering this evidence together, studying the biochemical pathways involved in the modulation of VEGF signaling might open up novel therapeutic strategies to prevent angiogenesis in arthritic cartilage and reduce disease progression.

Proteoglycans (PGs) are the main components of the ECM and provide structure and function. Interestingly, these molecules, by virtue of their ability to bind cytokines, chemokines, growth factors, and morphogens, regulate key biological functions, and their impaired regulation has been reported in arthritic articular chondrocytes [12,13]. 

Endocan is a small, soluble dermatan sulfate PG involved in inflammation, proliferation, and neovascularization processes [14,15,16]. The expression of such PG may be upregulated following the external administration of VEGF-A [17], inflammatory cytokines such as interleukin-1ß (IL-1ß) [18], and tumor necrosis factor-α (TNF-α) [19] by lipopolysaccharide (LPS) [20], and down-regulated by interferon-γ [16]. In fact, this PG is constitutively expressed in highly proliferative or neogenic tissues and cells; on the contrary, its expression is reduced or absent in quiescent tissues or resting cells [21]. In this setting, its increased expression may be considered a marker of endothelial activation and angiogenesis [21]. To note, endocan expression is not limited to the endothelial compartment because its expression has been reported in epithelial cells, cardiac muscle cells [21], synovial fibroblasts [22], and chondrocytes [18]. As a proangiogenic molecule, endocan functions are closely related to those of VEGF; in particular, it has been demonstrated that there is a positive feedback loop where VEGF-A positively regulates endocan expression, which in turn promotes VEGF-A signaling, ensuring its bioavailability and interaction with the vascular endothelial receptor 2 (VEGFR-2) [17,23]. Furthermore, it has been shown that endocan can regulate VEGF-A and VEGFR-2 expression, affecting the transcriptional factor NF-kB [24]. These data clearly show that endocan is simultaneously a target and a modulator of VEGF signaling, which promotes inflammation and angiogenesis [17,25,26,27]. 

Further, by engaging NF-kB, endocan regulates the expression of other key molecules, including matrix metalloproteinase 9 (MMP-9), whose expression has been correlated with increased VEGF-A expression [24]. In addition, in IL-1ß-inflamed synovial cells, endocan expression correlated with increased MMP-13 expression. On the contrary, endocan knockdown led to a down-regulation of such MMP, while the expression of MMP-1 and MMP-2 were not affected [22].

Endocan upregulation has been found in several pathological conditions, including cancer, chronic kidney disease, sepsis, systemic sclerosis, hypertension, and arthritis [22,28,29,30,31,32,33]. In arthritis, endocan expression has been correlated with the degree of inflammation in synovial tissues from patients with rheumatoid arthritis (RA) and osteoarthritis (OA), leading to the hypothesis that such PG may stimulate cell invasion, cell migration, and angiogenesis in arthritic joints [22].

In a previous study, we found that endocan was released by articular chondrocytes following IL-1ß induced inflammation in an NF-kB-dependent manner [18]. 

The expression of proangiogenic factors by resident cells represents a critical step in the mechanisms that promote the pathogenesis of arthritis [5]. As previously reported, in endothelial cells there is an interesting molecular interplay between endocan, VEGF-A, and VEGFR-2 with important consequences in the modulation of inflammation and angiogenesis [27].

Since inflammation and angiogenesis are two integrated processes in arthritis, we hypothesize that endocan, released by inflamed chondrocytes, could be involved in modulating pro-angiogenic factors. To elucidate this hypothesis in the present study, we investigated the effects of endocan knockdown on VEGF-A, VEGFR-2, MMP-9, and MMP-13 expression in a model of IL-1ß-induced inflammation in human articular chondrocytes. Furthermore, we investigated its effect on the activation of NF-kB.

## 2. Materials and Methods

### 2.1. Materials

Recombinant human IL-1β was purchased from Sigma–Aldrich s.r.l. (Milan, Italy). Human phosphor-VEGFR-2 polyclonal antibody was purchased from R&D Systems (Minneapolis, MN, USA). Flexi Tube GeneSolution, consisting of four pre-selected siRNAs for ESM-1 (endocan siRNA) to induce endocan knockdown, the negative control siRNA, and the transfection reagent (HiPerFect Transfection Reagent) were supplied by Qiagen (Hilden, Germany). Basal chondrocyte medium, fetal bovine serum (FBS), penicillin/streptomycin mixture, and glutamine were obtained from SCIENCELL™ (Carlsbad, CA, USA). Phosphate buffered saline solution (PBS) and other general laboratory chemicals were obtained from Sigma-Aldrich S.r.l. (Milan, Italy).

### 2.2. Cell Cultures

Human primary chondrocytes from articular cartilage were obtained from SCIENCELL^TM^ (Carlsbad, CA, USA). Cells were cultured in 75 cm^2^ plastic flasks containing 15 mL of the specific chondrocyte medium, to which 2.5% FBS, L-glutamine (2.0 mM); and penicillin/streptomycin (100 U/mL) were added. Cells were incubated at 37 °C in humidified air with 5% CO_2_. Experiments were performed using chondrocyte cultures between the third and the fifth passage.

### 2.3. Chondrocyte Treatment

Chondrocytes were cultured in six-well culture plates at a density of 2.5 × 10^5^ cells/well. Twenty-four hours after plating (time 0), the culture medium was replaced with OPTIMEM (Thermo Fisher Scientific, Waltham, MA, USA), and then cells were transfected with the endocan siRNA using the HiPerFect Transfection Reagent and following the manufacturer’s instructions. The negative control siRNA (NC siRNA) was used under the same conditions in other wells as a negative control. After 16 h, the medium was replaced with 2.0 mL of chondrocyte medium w/o antibiotics before the treatment with IL-1β (5 ng/mL). Finally, the cells and medium underwent biochemical evaluation 24 h later.

### 2.4. RNA Isolation, cDNA Synthesis, and qPCR Amplification

Total RNA was isolated from chondrocytes for qPCR evaluation of endocan, VEGF-A, MMP-9, MMP-13, and VEGFR-2 (mod. 7500, Applied Biosystems Inc., Carlsbad, CA, USA) using the TRIzol reagent kit (Thermo Fisher Scientific, Waltham, MA, USA). The first strand of cDNA was synthesized from 5.0 µg of total RNA using the high-capacity cDNA archive kit (Thermo Fisher Scientific, Waltham, MA, USA). β-Actin mRNA was used as an endogenous control to allow relative quantification. The amplified PCR products were quantified by measuring the calculated cycle thresholds (Ct) of endocan, VEGF-A, MMP-9, MMP-13, VEGFR-2, and β-actin mRNA. In addition, a melting curve analysis was always performed in order to verify the specificity of the reactions. After data normalization, using ß-actin as a housekeeping gene, the amount of specific mRNA in samples was calculated using the 2^−ΔΔCT^ method.

### 2.5. Protein Extraction and Western Blot Analysis

Protein extraction from cells was performed using a cell extraction buffer kit (Life Technologies, Carlsbad, CA, USA), which contained a protease inhibitor cocktail (Sigma-Aldrich, Milano, Italy), 1 nM phenylmethylsulfonyl fluoride (PMSF), and a phosphatase inhibitor cocktail (Sigma-Aldrich, Milano, Italy). A total of 20 µg of the protein extracted sample was mixed with Laemmli sample buffer with β-mercaptoethanol and separated on 12% SDS-polyacrylamide gels. Protein samples were blotted on PVDF membranes (Amersham Bioscience, Buckinghamshire, UK), using a specific transfer buffer. After blocking with TBS 0.1% Tween containing 5% non-fat dry milk for 1 h, membranes were incubated with a diluted (1:1000) mouse monoclonal antibody for ESM-1 (Abnova, Taipei, Taiwan) overnight at 4 °C. The next day, after three washes with TBS 0.1% Tween 20, the blots were incubated with a secondary peroxidase-conjugated goat anti-mouse antibody (Abcam, Cambridge, UK), diluted in TBS 0.15% Tween buffer, for 1 h at room temperature. Finally, the blots were washed five times with TBS 0.15% Tween 20, and the proteins were detected using a chemiluminescence (ECL plus) system (Amersham™, Stafford, UK). Images were obtained and quantified by scanning densitometry using a bio-image analysis system (C-DiGit, Li-cor, Lincoln, NE, USA). Results were expressed as relative integrated intensity using β-actin as an endogenous control (Cell Signaling, Danvers, MA, USA). 

### 2.6. Endocan, VEGF-A, MMP-9, and MMP-13 ELISA Assay

Samples of cell-secreted protein from the culture media, in the presence of 1 nM PMSF and a protease inhibitor cocktail, were centrifuged at 10,000× *g* at 4 °C for 10 min.

Endocan was detected using a commercial ELISA kit (RayBiotech, Peachtree Corners, GA, USA),

VEGF-A and MMP-9 levels were measured by commercially available ELISA kits provided by R&D Systems (Minneapolis, MN, USA), MMP-13 protein concentration by an ELISA kit provided by Abcam (Cambridge, UK), and all the assays were performed according to the manufacturer’s instructions. The absorbance of each sample was measured using a spectrophotometric microplate reader (DAS, Palombara Sabina Italy) set at λ450 nm. The results are expressed as pg/mL for endocan, VEGF-A, and MMP-13, and as ng/mL for MMP-9 protein levels. 

### 2.7. Immunofluorescence

The cells, seeded onto glass coverslips, were fixed in 4% paraformaldehyde in 0.2 M phosphate buffer (pH 7.4) for 2 h at room temperature (RT), and then they were washed 3 times for 10 min each with phosphate-buffered saline (PBS). To block nonspecific binding sites and to permeabilize the membranes, the cells were preincubated with 0.3% triton X-100 in PBS for 10 min and with 1% bovine serum albumin (BSA) in PBS for 30 min at RT. Then, cells were incubated with a rabbit anti phospho-VEGFR-2 polyclonal antibody (R&D Systems, Minneapolis, MN, USA) overnight at 4 °C. After numerous rinses, the primary antibody was detected using a FITC-conjugated IgG anti-rabbit (Jackosn ImmunoReseach Laboratories, Inc., West Grove, PA, USA) for 1 h at RT. Nuclei were stained with DAPI diluted 1:1000 in PBS for 10 min at RT. Finally, cells were washed in PBS, and the coverslips were mounted on slides. Immunofluorescence reactions were analyzed, and images were acquired using a Zeiss LSM 5 Duo (Carl Zeiss, Iena, Germany) confocal laser scanning microscope. All images were digitalized at a resolution of eight bits into an array of 2048 × 2048 pixels. Optical sections of fluorescence specimens were obtained using a HeNe laser (λ543 nm) at a 762-s scanning speed with up to eight averages. Contrast and brightness were established by examining the brightest-labeled pixels and choosing the settings that allowed clear visualization of the structural details while keeping the pixel intensity at its highest (200). Each image was acquired within 62 s, to minimize photodegradation, and five microscopic fields for each magnification were taken. Digital images were cropped, and figure montages were prepared using Adobe Photoshop 7.0 (Adobe System, Palo Alto, CA, USA).

### 2.8. NF-kB Activation

NF-kB activity was evaluated in cell lysates using the commercial ELISA kit NF-kB p65 (Phospho) [pS536] Human InstantOne™ ELISA Kit (Thermo Fisher Scientific, Waltham, MA, USA). This kit is specifically engineered for the measurement of phosphorylated (pS536) human p65. Briefly, 50μL of sample lysate, a negative control and a positive control were added to the plates and mixed with 50 μL of the antibody cocktail and incubated for 1h at room temperature. After washing, 100 μL of the detection reagent was added to each well and incubated within 20 min with shaking at 300 rpm. Finally, the reaction was stopped by adding 100 μL of stop solution. The absorbance of each sample was measured using a spectrophotometric microplate reader (DAS, Italy; IT) set at λ450 nm. Values are expressed as the relative optical density (OD) per mg protein.

### 2.9. Statistical Analysis

Data are expressed as the mean ± SD values of at least five experiments for each test. All assays were performed in duplicate to ensure reproducibility. Statistical analysis was performed using a one-way analysis of variance (ANOVA) followed by the Student–Newman–Keuls test. The statistical significance of differences was set at *p* < 0.05. Graphs were drawn using GraphPad Prism software (version 7.04 for Windows).

## 3. Results 

### 3.1. Endocan Expression Is Significantly Up-Regulated in IL-1ß Stimulated Chondrocytes

**Figure 1:**
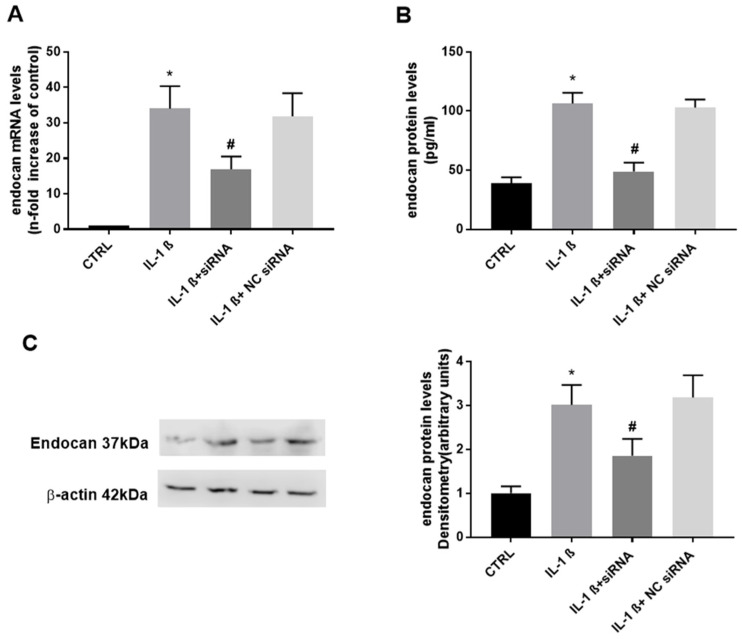
(**A**): endocan mRNA expression (relative fold change: CTRL = 1; IL-1ß = 34.031; IL-1ß + siRNA = 16.93; NC siRNA = 31.86). (**B**): endocan secreted protein (pg/mL: CTRL = 38.95; IL-1ß = 106.417; IL-1ß + siRNA = 48.91; NC siRNA = 102.91). (**C**): endocan intracellular protein (arbitrary units: CTRL = 1; IL-1ß = 3.014; IL-1ß + siRNA = 1.858; NC siRNA = 3.18). Data are the mean ± SD of five experiments. * *p* < 0.001 vs. CTRL; # *p* < 0.001 vs. IL-1ß.

Figure 1 shows the endocan expression levels in chondrocytes after treatment with IL-1ß for 24 h. As shown, IL-1ß induced a significant increase in both endocan mRNA and protein levels (Figure 1A–C). These increases were reduced in the presence of the endocan-specific siRNA. The NC-siRNA had no effect on the treatment. Thus, knocking down endocan using siRNA inhibited both its RNA expression and protein levels induced by IL-1ß. 

### 3.2. Endocan Knockdown Downregulates VEGF-A Expression in IL-1ß-Induced Chondrocytes

VEGF-A expresses only a minute amount under physiological conditions, whereas IL-1ß has been shown to induce VEGF-A in the chondrocytes [2,7].

**Figure 2:**
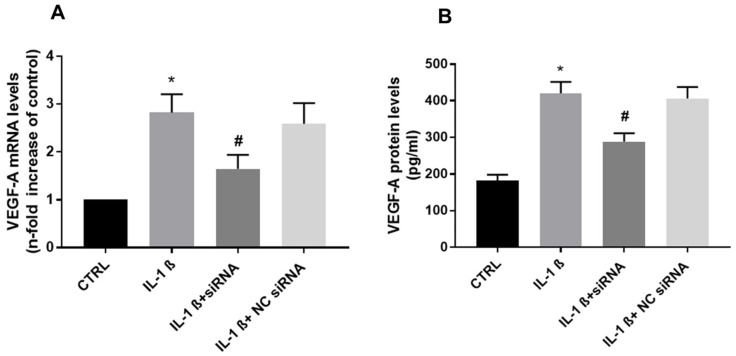
(**A**): VEGF-A mRNA expression (relative fold change: CTRL = 1; IL-1ß = 2.82; IL-1ß + siRNA = 1.64; NC siRNA = 2.58). (**B**): VEGF-A protein levels (pg/mL: CTRL = 181.87; IL-1ß = 420.41; IL-1ß + siRNA = 288.41; NC siRNA = 406.41). Data are the mean ± SD of five experiments. * *p* < 0.001 vs. CTRL; # *p* < 0.001 vs. IL-1ß.

Here, we examined the effect of IL-1ß on the expression of VEGF-A in chondrocytes. As shown in Figure 2, VEGF-A expression levels on both mRNA and protein were increased after IL-1ß treatment. Interestingly, the endocan-specific siRNA reduced that of the induction by IL-1ß, and there were no effects using the NC-siRNA in chondrocytes.

### 3.3. Endocan Knockdown Prevents MMP-9, MMP-13, and VEGFR-2 Expression in IL-1ß-Induced Chondrocytes

It has been shown that VEGF-A interacts with its receptor, VEGFR-2, which enhances MMP expression and mediates proteolysis of the ECM. This process contributes to new vessel formation [34].

**Figure 3:**
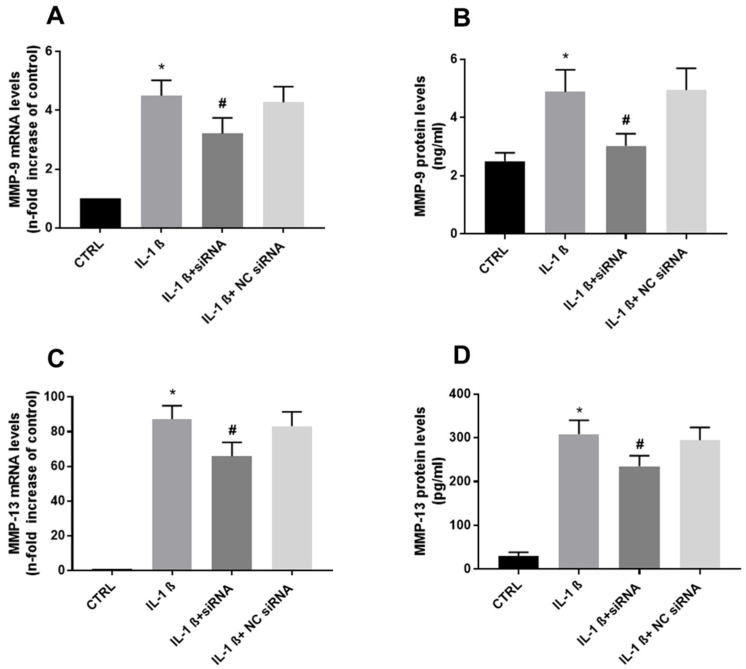
(**A**): MMP-9 mRNA expression (relative fold change: CTRL = 1; IL-1ß = 4.50; IL-1ß + siRNA = 3.21; NC siRNA = 4.27). (**B**): MMP-9 protein levels (ng/mL: CTRL = 2.50; IL-1ß = 4.88; IL-1ß + siRNA = 3.02; NC siRNA = 4.94). (**C**): MMP-13 mRNA expression (relative fold change: CTRL = 1; IL-1ß = 87.3; IL-1ß + siRNA = 65.83; NC siRNA = 83.29). (**D**): MMP-13 protein levels (pg/mL: CTRL = 30.33; IL-1ß = 308.33; IL-1ß + siRNA = 235; NC siRNA = 294.5) Data are the mean ± SD of five experiments. * *p* < 0.001 vs. CTRL; # *p* < 0.001 vs. IL-1ß.

Studies have shown that endocan modulates the expression of MMP-9 and MMP-13 [22,23,35], we thought then, to examine the effect of endocan expression on MMPs in chondrocytes. As shown in Figure 3, treating chondrocytes with IL-1ß significantly increased the expression of both MMP-9 and MMP-13. Similarly, the increases were significantly inhibited by adding endocan-specific siRNA but not by NC-siRNA treatment. Thus, our results demonstrated that endocan regulates both MMP-9 and MMP-13 expression.

**Figure 4:**
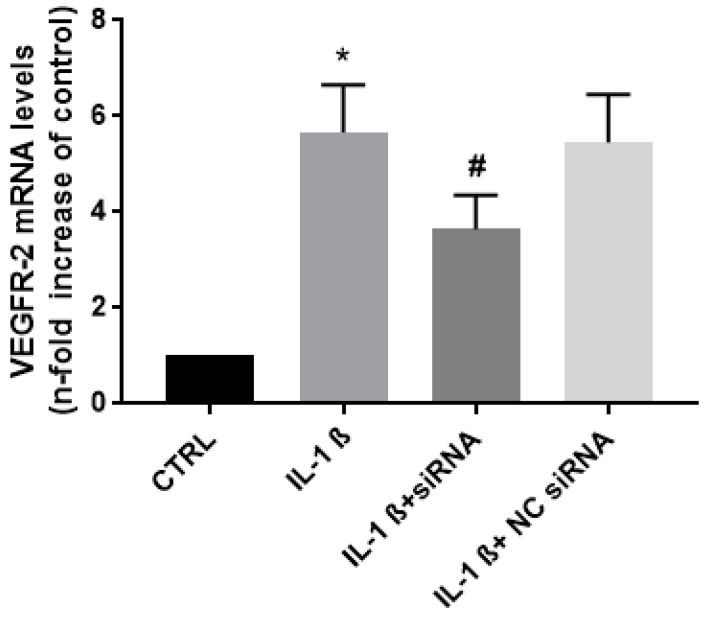
VEGFR-2 mRNA expression in IL-1ß activated chondrocytes in the presence/absence of the endocan siRNA. Relative fold change: CTRL = 1; IL-1ß = 5.66; IL-1ß + siRNA = 3.64; NC siRNA = 5.44. Data are the mean ± SD of five experiments. * *p* < 0.001 vs. CTRL; # *p* < 0.001 vs. IL-1ß.

Next, we examined the effect of endocan on the expression of the VEGFR-2 receptor. The results show that IL-1ß increased the VEGFR-2 mRNA levels six-fold, compared to the control. The treatment of cells with endocan-specific siRNA, reduced this induction to four-fold, whereas no effect on NC-siRNA-treated cells (Figure 4).

**Figure 5:**
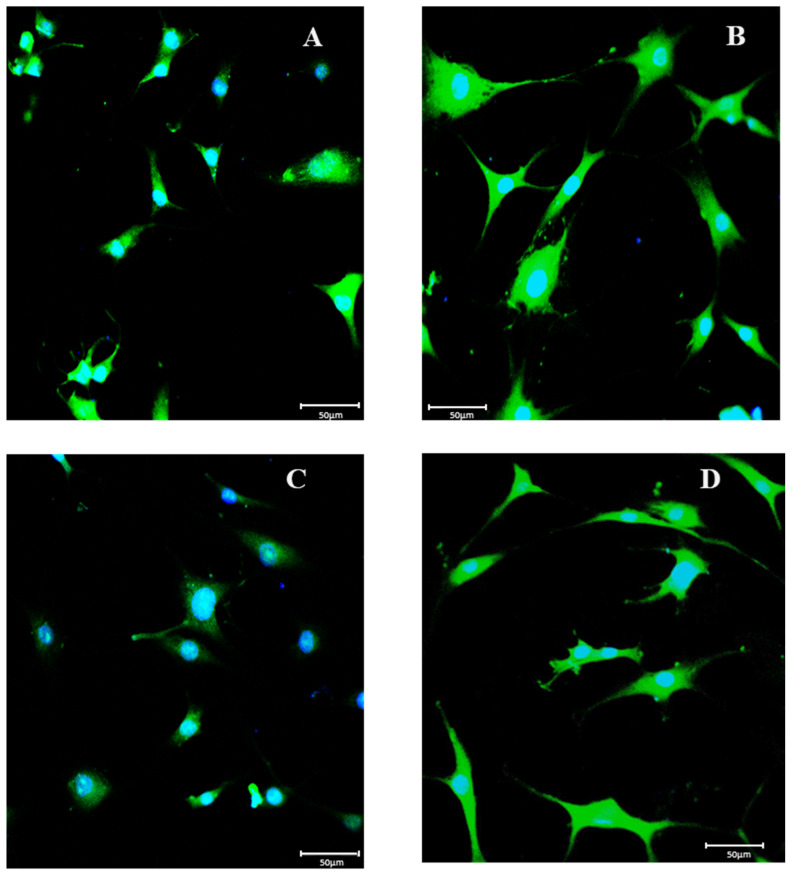
Compound panel of immunofluorescence reactions using an anti-pVEGFR-2 antibody (green fluorescence). The control cells exhibited the presence of a pVEGFR-2 staining pattern around the nuclei and at the plasma membrane level (**A**); in chondrocytes treated with IL-1ß, the signal was increased in the perinuclear zone, in the plasma membrane, and at the cellular extension level (**B**); in chondrocytes, where endocan knockdown was induced, the p-VEGFR-2 fluorescence was strongly reduced (**C**), the treatment with the NC siRNA did not modify IL-1ß effects (**D**). Images were acquired at a magnification of 40×.

Furthermore, we investigated the p-VEGFR-2 pattern of expression by immunofluorescence. As shown in Figure 5, control cells exhibited the presence of the VEGFR-2 staining pattern; it seems to surround the nuclei and also to be at the plasma membrane level (A). The fluorescence pattern was significantly increased in IL-1ß-stimulated chondrocytes (B); the signal was increased in the perinuclear zone, in the plasma membrane, and at the cellular extension level when compared to the control cells. On the contrary, endocan knockdown chondrocytes stimulated with IL-1ß showed a strong reduction of the VEGFR-2 fluorescence pattern when compared to normal and IL-1ß stimulated chondrocytes (C), while the treatment with the NC siRNA did not modify IL-1ß effects (D).

Taken together, our results suggest that endocan might play a role in disrupting the interaction of VEGF-A with its receptor, VEGFR-2, which affects the expression of MMP-9 and MMP-13.

### 3.4. Endocan Knockdown Prevents NF-kB Activation in IL-1ß-Induced Chondrocytes

**Figure 6:**
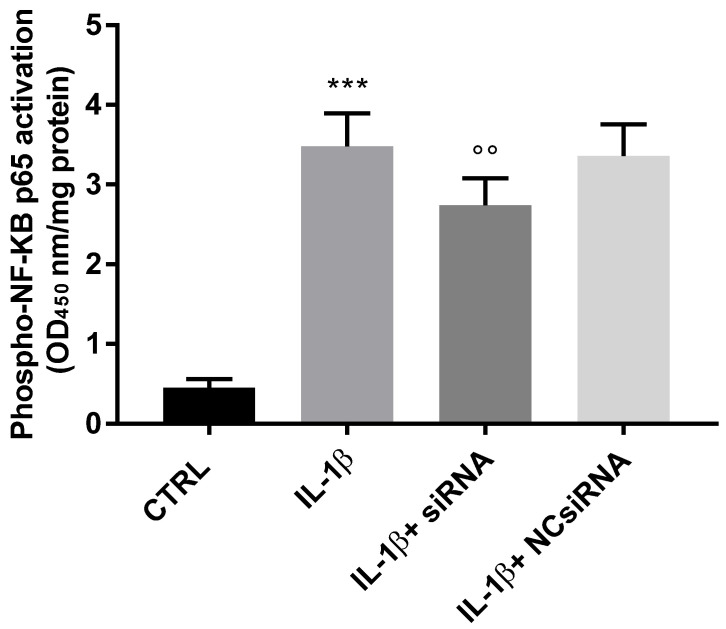
NF-kB p65 degree of phosphorylation (pS536) in IL-1ß activated chondrocytes in the presence/absence of the endocan siRNA. OD_450_ nm/mg protein: CTRL = 0.45; IL-1ß = 3.48; IL-1ß + siRNA = 2.74; NC siRNA = 3.36). Data are the mean ± SD of five experiments. *** *p* < 0.001 vs. CTRL; °° *p* < 0.01 vs. IL-1ß.

Studies have demonstrated that endocan modulates NF-kB activity to promote the expression of many genes involved in inflammation and angiogenesis [18,24]. Herein, we used a commercial ELISA kit to measure the NF-kB p65 activity in cells treated with IL-1ß with or without endocan-specific siRNA. The activity was shown as OD450 nm/mg protein in Figure 6. There was a significant increase in p65 activity in cells treated with IL-1ß compared to the controls. This increment was inhibited 0.21-fold, but significantly, after the treatment with the endocan-specific siRNA, but not with that of the NC-siRNA treatment. Thus, endocan probably affects the activity of NF-kB.

## 4. Discussion

In the present study, we investigated the effects of endocan knockdown on the expression of pro-angiogenic molecules during IL-1ß induced inflammation in human articular chondrocytes. Furthermore, we studied these effects on NF-kB activation to evaluate the involvement of such PG in the modulation of this signaling pathway.

Endocan is a small soluble DSPG previously identified as an endothelial-specific PG, regulated by cytokines and pro-angiogenic molecules and involved in endothelial cell adhesion, migration, proliferation, angiogenesis, and inflammation [16,36]. This PG is constitutively secreted in active and neogenic tissues and absent in silent cells or reposing tissues [21,37]. In a previous study, we found that endocan was expressed by articular chondrocytes during IL-1ß-induced inflammation due to IL-1ß induced NF-kB activation [18]. Furthermore, Kim et al. reported an increased expression of such PG and VEGF in the inflammatory sites of patients with RA and OA. In OA patients, they found that endocan expression was higher in tissue with moderate and severe inflammation than in those with mild inflammation. Interestingly, they also found that endocan knockdown inhibits the invasive and migratory phenotype of IL-1ß-inflamed synovial fibroblasts, suggesting a role of such PG in cell invasion and migration, and increased angiogenesis in arthritic joints [22].

Angiogenesis is a fine-tuned mechanism that consists of the formation of new capillary blood vessels from existing vasculature [1]. This process occurs in physiological conditions, such as embryogenesis and wound healing, and in pathological conditions, such as cancer and arthritis [1,38].

In the context of arthritis, activated chondrocytes participate actively in this process because of their ability to produce and release pro angiogenic molecules and protolithic enzymes. These factors, acting in an autocrine and paracrine manner, mediate endothelial cells infiltration and cartilage degradation [10].

Our data have shown that endocan, VEGF-A, MMP-9, MMP-13, and VEGFR-2 expression was increased in articular chondrocytes stimulated with IL-1ß. On the contrary, when endocan expression was knocked down by the specific siRNA before IL-1ß addition, the expression of such parameters was significantly reduced. Furthermore, we found that the downregulation of such targets correlated with reduced pVEGFR-2 protein expression and NF-kB activation.

As reported, the pro-angiogenic factor VEGF-A and its receptor VEGFR-2 are expressed during the early stage of cartilage development. On the contrary, their expression is suppressed in mature chondrocytes from adult cartilage [2]. However, in the presence of a *noxa*, the resulting hypertrophic chondrocytes re-express VEGF-A and VEGFR-2, mediating inflammation, and pathological angiogenesis [2]. As expected, we found that VEGF-A and VEGFR-2 were significantly expressed after IL-1ß stimulation; interestingly, their expression was significantly affected by endocan knockdown. The same effects were seen when evaluating pVEGFR-2 protein expression.

These findings, in accordance with previously published data [27], clearly demonstrated that endocan could modulate VEGF-A and VEGFR-2 expression also in IL-1ß chondrocytes.

MMPs are a family of zinc-dependent metalloendopeptidases that play a critical role in the turnover of the ECM and their deregulation has been reported in arthritic joints in both OA and RA [39,40,41].

In our study, we investigated the expression of MMP-9 and MMP-13, two MMPs that have been correlated with endocan expression and function [22,24].

Concerning the link between endocan and MMP-9, it has been shown that endocan knockdown reversed MMP-9 increased expression in radiotherapy-resistant breast cancer cells; on the contrary, MMP-9 and also VEGF-A expression was significantly improved in mice overexpressing endocan [24]. In addition, such effects were also confirmed in a study on the role of endocan in mediating pathological ocular angiogenesis in mice [23]. The MMP-9 data obtained in the present study support these previous results, indicating a biochemical connection between endocan expression and MMP-9 up-regulation.

As for MMP-9, it has been shown that there is a positive correlation between MMP-13 and VEGF-A/VEGFR-2 expression and vascular angiogenesis [41,42,43]. Despite MMP-9, the effects of endocan on MMP-13 expression were poorly investigated. However, in the context of arthritis, Kim et al. have shown that endocan knockdown down-regulated the expression of such metalloproteinase in synovial fibroblasts inflamed by IL-1ß [22]. In line with these previously reported findings, we found the same effects in IL-1ß inflamed chondrocytes where endocan was knocked down.

The transcription factor nuclear factor-κB (NF-κB) modulates an array of genes that regulate inflammatory responses, cell proliferation, tumorigenesis, cell survival, and development. Because of these functions, the deregulation of such transcription factor activity has been reported in several pathological conditions, including cancer, autoimmune diseases, and arthritis [44].

In RA, the release of inflammatory cytokines and autoantibodies by inflammatory cells continuously activates the NF-kB pathway in the synovial tissues of affected joints [45].

In OA, the same effects are observed, in which, following inflammation and mechanical loading, the NF-kB pathway is constitutively activated and participates in regulating many pathological events, including chondrocyte catabolism, chondrocyte survival, and synovial inflammation [44]. In this setting, NF-kB upstream regulators, co-factors, and downstream effectors represent potential targets to prevent both RA and OA progression [44,45].

The ability of endocan to regulate NF-kB activity has been reported in colorectal cancer [46], hepatocarcinoma [47], and leukemia [48]. In our study, we found that the activation of such transcription factor induced by IL-1ß treatment was significantly affected by endocan knockdown.

**Figure 7:**
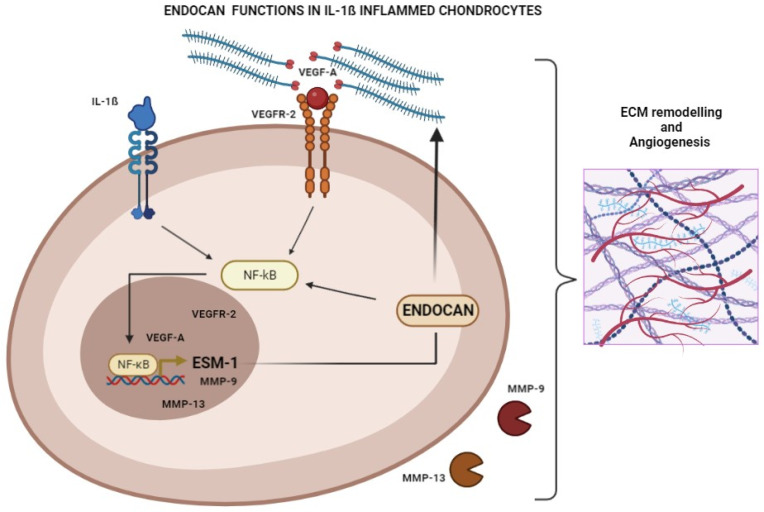
Endocan functions in IL-1ß inflamed chondrocytes: IL-1ß activates NF-kB, promoting the expression of endocan, VEGF-A, VEGFR-2, MMP-9, and MMP-13. Endocan, in turn, participates in this signaling, in part by facilitating the VEGF-A/VEGFR-2 interaction and, in part, by promoting their expression, modulating NF-kB activity. These functions lead to the hypothesis that endocan may promote angiogenesis in inflamed cartilage. (Figure was drawn using the web-based tool BioRender: https://biorender.com).

Taken together, these findings allow us to suggest a model where IL-1ß activates NF-kB and enhances the expression of VEGF-A, MMP-9, MMP-13, VEGFR-2, and endocan. Released endocan, in turn, participates in this signaling, in part by facilitating the VEGF-A/VEGFR-2 interaction and in part by promoting their expression, modulating NF-kB activity. In fact, when endocan was knocked down, the VEGF-A, MMP-9, MMP-13, and VEGFR-2 expression and NF-kB activation were significantly decreased (Figure 7).

In conclusion, we have highlighted the importance of endocan in inflamed articular chondrocytes as a key molecule able to modulate proangiogenic factors expression and NF-kB activity. These results lead to the hypothesis that this PG may be involved in the mechanisms that attend to new vessel formation in arthritic cartilage. However, further studies are needed to better clarify the exact mechanisms by which endocan mediates these effects and its role in the arthritic process.

## Data Availability

The data presented in this study are available on request from the corresponding author.
